# Genetic dissection of yield traits in super hybrid rice Xieyou9308 using both unconditional and conditional genome-wide association mapping

**DOI:** 10.1038/s41598-017-00938-7

**Published:** 2017-04-11

**Authors:** Yingxin Zhang, Liyuan Zhou, Xihong Shen, Daibo Chen, Weixun Wu, Xiaodeng Zhan, Qunen Liu, Aike Zhu, Xiangyang Lou, Haiming Xu, Shihua Cheng, Liyong Cao

**Affiliations:** 1 State Key Laboratory of Rice Biology and Zhejiang Key Laboratory of Super Rice Research, China National Rice Research Institute, Hangzhou, 311401 China; 2 Institute of Crop Science and Institute of Bioinformatics, College of Agriculture and Biotechnology, Zhejiang University, Hangzhou, 310058 China; 3 Sir Run Run Shaw Hospital, School of Medicine, Zhejiang University, Hangzhou, 310058 China; 4 Department of Pediatrics, Biostatistics Section, Arkansas Children’s Hospital Research Institute School of Medicine, University of Arkansas for Medical Sciences, Little Rock, AR 72202 USA

## Abstract

With the development and application of super rice breeding, elite rice hybrids with super high-yielding potential have been widely developed in last decades in China. Xieyou9308 is one of the most famous super hybrid rice varieties. To uncover the genetic mechanism of Xieyou9308’s high yield potential, a recombinant inbred line (RIL) population derived from cross of XieqingzaoB and Zhonghui9308 was re-sequenced and investigated on the grain yield (GYD) and its three component traits, number of panicles per plant (NP), number of filled grains per panicle (NFGP), and grain weight (GW). Unconditional and conditional genome-wide association analysis, based on a linear mixed model with epistasis and gene-environment interaction effects, were conducted, using ~0.7 million identified SNPs. There were six, four, seven, and seven QTSs identified for GYD, NP, NFGP, and GW, respectively, with accumulated explanatory heritability varying from 43.06% to 48.36%; additive by environment interactions were detected for GYD, some minor epistases were detected for NP and NFGP. Further, conditional genetic mapping analysis for GYD given its three components revealed several novel QTSs associated with yield than that were suppressed in our unconditional mapping analysis.

## Introduction

Rice is a fundamentally important staple crop, and improving rice yields has remained a major goal in world agriculture. Super hybrid rice shows great advantages in grain yield and biomass in comparison with conventional rice varieties. Since its inception in China in 1996, super rice breeding program has achieved tremendous increases in rice yields^1^. Xieyou9308 is one of the most famous super hybrid rice varieties with a grain yield as high as 12.23 t/ha^1^. However, the genetic basis underlying this high yield potential remains largely unclear. In order to fuel the further successes of super rice breeding programs, continued efforts to dissect the genetic basis of economically-important traits will be necessary.

Economically, the most important agronomic trait for rice is grain yield (GYD). GYD exhibits complex genetics, as it is known to be an integrated quantitative trait that is influenced variously by yield component traits and by the environment. Several QTL linkage mapping studies with Xieyou9308 have used conventional molecular markers to explore the causal loci that are responsible for the phenotypic variation of economically-important traits^2–4^. However, owing to the insufficient density of polymorphism markers, the QTLs reported in these studies could typically only be localized to very large chromosomal regions, where still may harbor considerable amounts of genetic variants^5^. This restricts the consequent application of these QTLs in marker assisted breeding to some extent.

Partly impelled by advances in sequencing technologies and the resulting improvements in genotyping, genome-wide association study (GWAS) strategy has become one of the primary approaches used to identify causal genes underlying phenotypic variation. GWAS is particularly attractive because it offers hope for rapidly narrowing the region where a causal gene might lie. Although pioneered by human geneticists, GWAS is also being appealingly applied to plants including rice^6–12^. Huang *et al*.^9^ re-sequenced 517 rice landraces and used GWAS methods to analyze putative causal relationships between 14 agronomic traits and ~3.6 million SNPs, from which they identified three loci associated with tiller number, two loci associated with spikelet number, two loci associated with grain width, and five loci associated with grain length. A subsequent study from Huang *et al*.^12^ reported 32 new loci associated with 11 agronomic traits based on a natural population of 950 worldwide rice varieties. Another GWAS based on 413 diverse accessions of O. *sativa* from 82 countries identified 234 loci associated with 34 agronomic traits using 44,100 identified SNP variants^11^.

These studies confirm that GWAS is a powerful approach that can be used in rice to identify genetic variants associated with complex traits with high resolution. However, most of these studies were focused on detecting genetic variant exhibiting additive genetic effects without consideration of gene-environmental and gene-gene interactions which were thought to be very important for complex traits. In addition, the cryptic population structure in the rice natural population (collected landraces) which would increase the false positive associations also haunted the researchers. Moreover, although increasing numbers of association studies have attempted to map the casual genes for yield traits of rice, most of these studies dissected traits separately, without considering genetic correlations between traits. As yield traits are known to be interrelated, exploring genetic correlations among these traits should provide additional insights into the genetic basis of grain yield. Conditional genetic analysis is a methodology first introduced by Zhu^13^ to study developmental quantitative genetics; it was later extended for the analysis of the genetic contributions of component traits to an integrated trait at the molecular level^14, 15^.

In this study, the derived recombinant inbred line (RIL) population of Xieyou9308, which should theoretically have no deleterious issues relating to population structure, were re-sequenced and used for both genome-wide association mapping and for conditional association mapping for GYD and its three constitutive traits. The analysis was based on a saturated mixed linear model that included both epistasis and gene-environmental interactions. Further, a conditional methodology was adopted to identify additional candidate regions that likely contribute to grain yield. Our results provided some information that should be of use in efforts seeking genetic improvement of yield potential in rice.

## Results

### Phenotypic variation of yield traits and their inter-correlations

**Figure 1:**
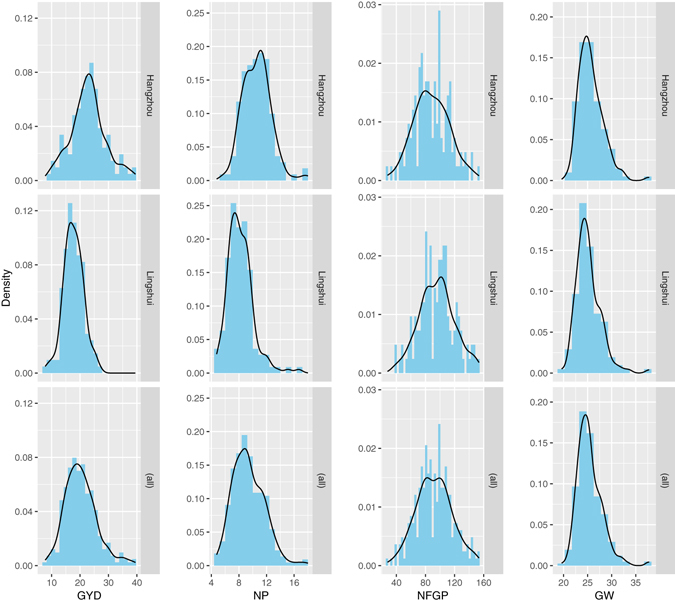
The phenotypic distribution of grain yield and yield components. The histograms for phenotypes from each experiment location Hangzhou (top panel), Lingshui (middle panel), and their together (bottom panel) were plotted; and each column represents one phenotype (from left to right: grain yield (GYD), number of panicles (NP), number of filled grains per panicle (NFGP), grain weight (GW)).

As shown in Table 1, all four traits varied widely among the RI lines (CV = 9.88~27.51% in E1, and 10.23~25.36% in E2), with the NFGP trait showing the largest variation and the GW trait showing the smallest variation for both locations. Additionally, significant differences in mean values were detected at the 0.05 significance level (Tukey’s test) for the GYD, NP, and NFGP traits between the two environments. All four traits segregated continuously (Fig. 1), and the NP and NFGP exhibited approximately bimodal distributions, probably suggesting the existence of complex genetic bases underlying these two traits. The inter-correlations in phenotypic values and genotypic values between any two of the four traits are presented in Table 2. Significant positive correlations were observed between GYD and its three components: GYD had relatively higher positive correlations with NFGP than with NP, and insignificant positive correlation with GW. In contrast, the component traits were negatively correlated with each other. There was an especially strong negative correlation between NP and NFGP, which indicated that it will likely be necessary to conduct conditional mapping as the variation caused by these two components could be counteracted by the opposite effects during the formation of the final yield trait.

**Table 1:** Summary statistics of grain yield and yield components in two experiment locations.

Trait	Hangzhou			Lingshui			D
	Mean ± SD	Range	CV(%)	Mean ± SD	Range	CV(%)	
GYD	22.86 ± 6.10	8.50–39.50	26.68	17.69 ± 3.47	7.70–27.00	19.61	5.17**
NP	10.60 ± 2.03	5.60–18.00	19.2	8.21 ± 1.86	4.80–16.70	22.63	2.38*
NFGP	88.22 ± 24.27	27.8 0–152.50	27.51	95.69 ± 24.26	38.10–153.60	25.36	7.47**
GW	25.68 ± 2.54	20.7 0–37.60	9.88	25.14 ± 2.57	19.60–37.80	10.23	0.54

GYD = grain yield; NP = number of panicles per plant; NFGP = number of filled grains per panicle; GW = grain weight; CV = coefficient of variation; D = the difference in mean between two experimental locations; *p ≤ 0.05; **p ≤ 0.01.

**Table 2:** Phenotypic and genotypic correlations for four yield traits.

Trait	GYD	NP	NFGP	GW
GYD		0.17*	0.61***	0.10
NP	0.23**/0.20*		−0.53***	−0.17*
NFGP	0.68***/0.52***	−0.37***/−0.57***		−0.16
GW	0.09/0.12	−0.13/−0.15	−0.15/−0.15	

The up-triangle are genotypic correlation coefficients estimated by predicted genotypic values; the first and the second values in the low-triangle are phenotypic correlation coefficients for the environment 1 (Hangzhou) and 2 (Lingshui), respectively; *, **, *** indicate the significant level of 0.05, 0.01 and 0.005; GYD = grain yield, NP = number of panicles per plant, NFGP = number of filled grains per panicle, GW = grain weight.

### Genome-wide association analyses for four yield traits

**Figure 2:**
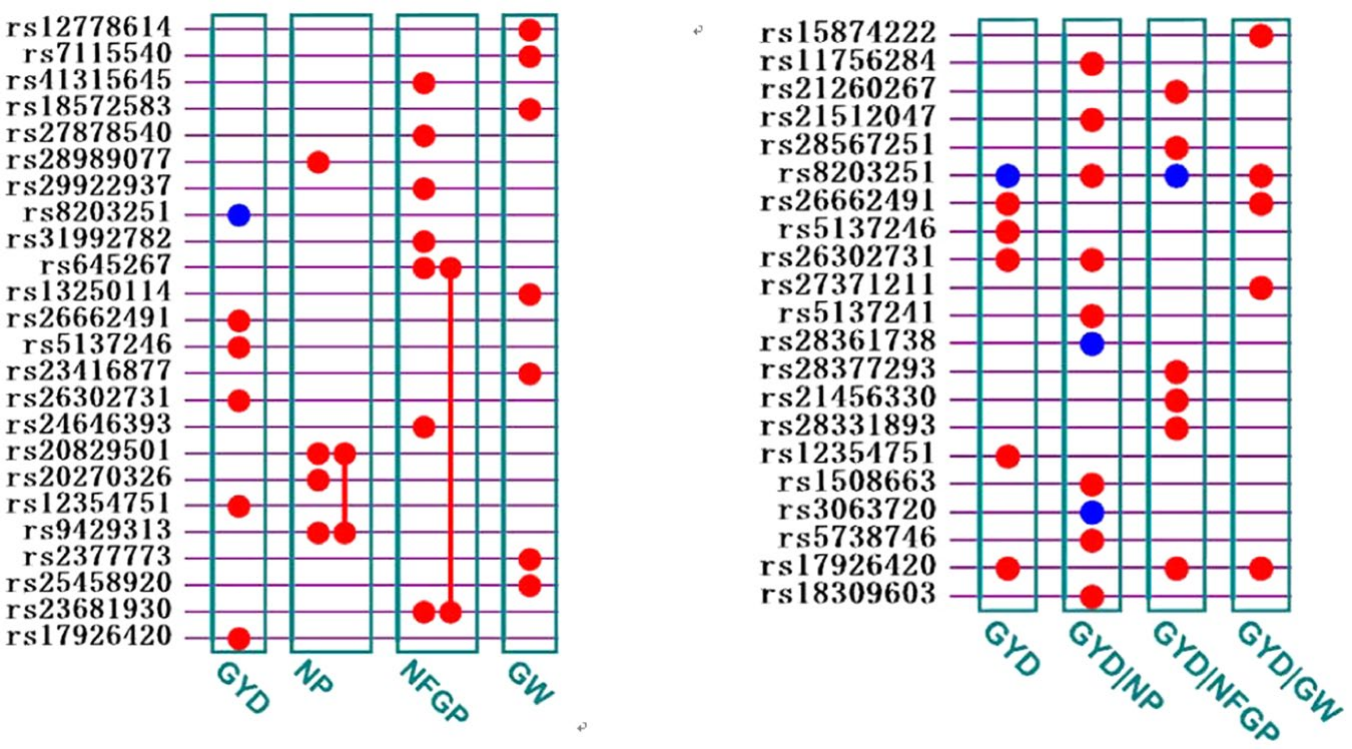
Genetic architecture of detected QTSs for grain yield and yield components in both the unconditional mapping and conditional mapping analyses. Circle = QTX individual additive effect; Line between two QTXs = epistasis effects; Red = general effects across two environments; Blue = general and environment-specific effects.

In total, there were 24 SNPs detected significantly (6 SNPs for GYD, 4 SNPs for NP, 7 SNPs for NFGP, and 7 SNPs for GW) with accumulated explanatory heritabilities varying from 43.06% to 48.36%. There was an additive by environment interaction detected in grain yield (GYD), and some epistatic effects were detected for NP and NFGP (Table 3; Fig. 2).

**Table 3:** The estimated heritability and predicted genetic effects of all detected significant SNP loci for four yield traits.

Trait	QTS	Chr.	Allele	Effect type	Effect size	−*log*_10_(*P*)	*h* ^2^(%)	${{\boldsymbol{h}}}_{{\boldsymbol{T}}}^{{\boldsymbol{2}}}$(%)
GYD	rs8203251	4	C/T	a	−1.54	12.43	10.91	43.06
				ae1	−0.65	1.62	1.91	
				ae2	0.64	1.61		
	rs26662491	5	A/C	a	−1.04	6.09	5.02	
	rs5137246	6	T/G	a	1.19	7.76	6.56	
	rs26302731	6	G/A	a	−1.42	10.75	9.34	
	rs12354751	9	A/G	a	−0.93	4.98	4.01	
	rs17926420	12	C/A	a	−1.07	6.4	5.31	
NP	rs28989077	3	C/T	a	−0.83	20.65	18.54	43.35
	rs20829501	7	C/T	a	−0.65	13.01	11.39	
	rs20270326	9	T/C	a	−0.44	6.23	5.12	
	rs9429313	10	A/C	a	−0.47	7.16	5.98	
	rs20829501 & rs9429313	7 & 10	C/T & A/C	aa	0.29	3.12	2.32	
NFGP	rs41315645	1	G/A	a	6.31	9.7	8.16	44.40
	rs27878540	3	C/T	a	4.31	4.87	3.82	
	rs29922937	3	A/T	a	6.45	10.1	8.53	
	rs31992782	4	A/T	a	−4.6	5.45	4.33	
	rs645267	5	A/G	a	−5.57	7.7	6.35	
	rs24646393	6	G/A	a	4.23	4.7	3.67	
	rs23681930	11	C/T	a	5.83	8.38	6.96	
	rs645267 & rs23681930	5 & 11	A/G & C/T	aa	3.55	3.47	2.58	
GW	rs7115540	1	C/T	a	−0.49	7.09	5.43	48.36
	rs12778614	2	A/T	a	−0.44	5.93	4.45	
	rs18572583	3	T/C	a	−0.82	18.52	15.19	
	rs13250114	5	A/C	a	−0.5	7.4	5.69	
	rs23416877	6	G/A	a	−0.54	8.64	6.74	
	rs2377773	11	C/T	a	−0.46	6.43	4.87	
	rs25458920	12	T/C	a	0.51	7.76	5.99	

QTS = the detected significant SNPs associated with the yield and yield components traits; Chr. = chromosome; Allele = paternal allele/maternal allele; a = additive effect for paternal allele homozygotes (QQ, ZH9308), ae = additive by environmental interaction effect, aa = additive by additive epistasis effect; −*log*_10_(*P*) = inverse of the base 10 logarithm of p value; *h* ^2^(%) = heritability in percentage due to the genetic component effect; ${h}_{T}^{2}$(%) = total heritability equal to summation of heritabilities of all individual QTSs; GYD = grain yield, NP = number of panicles per plant, NFGP = number of filled grains per panicle, GW = grain weight.

As shown in Table 3, for GYD, there were 6 significant SNPs located on 4 chromosomes, together accounting for 43.06% of the phenotypic variation. All QTSs except one (rs5137246 on chromosome 6), showed large negative additive effects with individual contributions to the heritability in a range from 4.01% to 10.91%. The negative additive genetic effects indicated that the paternal homozygous genotype (QQ, Q here referred to as the allele from ZH9308) would decrease the grain yield, while the corresponding maternal allele homozygotes (qq) would increase the grain yield. The QTS located on chromosome 4 (rs8203251) exhibited the largest main additive (*h* ^2^ = 10.91%), and additive by environment interaction which showed opposite genetic effects in the two different locations. For NP, the total heritability (43.35%) mainly consisted of additive heritability (41.03%) from 4 QTSs whose individual heritability was quite large, especially for rs28989077 (*h* ^2^ = 18.54%). The remaining heritability was from one pair of additive by additive epistasis effects (rs20829501/rs9429313). All the main additive effects were negative; only one epistasis was positive, and its genetic effect size was relatively small as compared with the main additive effects (Table 3).

For NFGP, unlike the aforementioned two traits, most detected QTSs showed positive and modest-size additive effects, suggesting that the paternal-allele homozygotes (QQ) in these detected SNP loci would increase the number of filled grains. Additionally, there was a pair of positive epistasis interactions (rs645267/rs23681930) detected; and their individual main additive exhibited opposing genetic effects (rs645267 was negative while rs23681930 was positive). For GW, 7 SNPs, all with only additive effects, were found; in aggregate, these QTSs accounted for 48.36% of phenotypic variation. Of particular note, the SNP located on chromosome 3 (rs18572583) contributed 15.19% of the phenotypic variation and should thus be considered to be a very important candidate locus for subsequent breeding efforts.

### Conditional association mapping for GYD given its component trait

All significant QTSs with additive effects for yield conditioned on each of its components are presented in Table 4. There were 10, 7, and 5 QTSs detected, respectively, for yield conditioned on number of panicles (GYD|NP), yield conditioned on number of filled grains per panicle (GYD|NFGP), and yield conditioned on grain weight (GYD|GW). For GYD|NP, two QTSs (rs8203251 and rs26302731) were remained to be detected and eight novel conditional QTSs were identified in comparison with unconditional yield mapping (Tables 3 and 4). For the two overlapping QTSs, rs26302731 was supposed to be independent upon NP, as there was no significant difference in additive effects in the 95% confidence interval between unconditional and conditional mapping (data not shown), while rs8203251 was supposed to be involved in variation of NP as the additive by environment interaction failed to be detected in the conditional mapping (Fig. 2), even though the additive main effect was still similar. The eight novel conditional QTSs exhibited modest-sized effects, with individual contributions to phenotypic variation ranging from 2.31% to 6.58%. Similar results were observed for GYD|NFGP; most detected QTSs (5 out of 7) were novel loci that were supposed to be suppressed by the given component trait in the unconditional mapping results. This might be true because the conditional analysis could exclude the impact of the given component trait on the target trait and thus reveal the genes masked by an antagonist or repressor in the component trait. A different phenomenon was observed in GYD|GW mapping that only a small number of additional QTSs (2 SNPs) which were supposed to be suppressed by grain weight was detected. The small number of novel QTSs in the GYD|GW mapping may mainly result from the counterbalance between the two remaining components and partly from the relatively weak correlation between grain yield and grain weight. These results at the molecular level showed good agreement with those obtained using genotypic or phenotypic correlation analysis.

**Table 4:** The estimated heritability and predicted genetic effects of all detected SNP loci for grain yield conditioned by its component traits.

QTS	Chr.	Allele	GYD|NP			GYD|NGFP			GYD|GW		
			Effect size	−*log*_10_(*P*)	*h* ^2^(%)	Effect size	−*log*_10_(*P*)	*h* ^2^(%)	Effect size	−*log*_10_(*I*)	*h* ^2^(%)
rs8203251	4	C/T	−1.25	12.09	9.01	−0.59	3.02	2.52	−1.73	7.6	8.28
rs26302731	6	G/A	−0.88	6.29	4.43						
rs17926420	12	C/A				−0.8	5.18	4.7	−1.59	6.57	7.05
rs26662491	5	A/C							−1.05	3.15	3.05
rs5137246	6	T/G									
rs12354751	9	A/G									
rs11756284	2	T/G	−0.69	4.06	2.71						
rs21512047	2	C/T	−0.96	7.45	5.34						
rs28361738	6	G/A	−0.96	7.39	5.29						
rs5137241	6	T/C	0.79	5.24	3.61						
rs1508663	10	T/G	−0.79	5.18	3.57						
rs3063720	10	G/A	−0.63	3.54	2.31						
rs5738746	10	G/A	−1.07	9.03	6.58						
rs18309603	12	A/G	−0.97	7.52	5.39						
rs21260267	2	C/T				−0.96	7.16	6.74			
rs28567251	3	T/C				−0.77	4.87	4.39			
rs28377293	6	G/C				−0.93	6.7	6.27			
rs21456330	7	T/C				−0.77	4.82	4.33			
rs28331893	7	A/C				−0.77	4.83	4.35			
rs15874222	2	T/A							1.18	3.87	3.88
rs27371211	6	G/A							−1.23	4.17	4.23

QTS, Chr., Allele, −*log*_10_(*P*), *h* ^2^(%), GYD, NP, NFGP, GW have same definitions as those in Table 3.

Comparing the results of the unconditional and conditional association mapping for GYD (Table 4 and Fig. 2), three possible scenarios were evident : (1) An unconditional QTS was still detected under conditional mapping, generally with some fluctuation in genetic effect size, indicating this particular QTS was independent of (i.e., no significant difference in genetic effect size between unconditional and conditional mapping), or was partially correlated with, the corresponding given component trait; (2) A previously-detected unconditional QTS was totally absent under conditional mapping, suggesting that this QTS may be associated with the final grain yield through the corresponding given conditional component trait; (3) Some novel QTSs were detected under conditional mapping, suggesting that these QTSs might have been suppressed by the corresponding given component trait in the unconditional mapping analysis. As shown in Table 4, the rs8203251 locus was the only locus detected in the unconditional mapping and in all three of the conditional mappings; it seemed to be partially-correlated with NP and NFGP but independent of GW, as the difference in genetic effects was not significant (data not shown). Three QTSs were detected both in the unconditional mapping and at least one conditional mapping: rs26302731 was independent of NP but had high correlations with both NFGP and GW; rs17926420 was independent of both NFGP and GW but was highly correlated with NP; rs26662491 was independent of GW but had high correlations with both NP and NFGP. Two QTSs, rs5137246 and rs12354751, were totally absent in the three conditional mappings, suggesting they were highly correlated with the three component traits simultaneously. There was also a batch of novel QTSs associated with GYD that were detected through different conditional mappings. As shown in Table 4, in general, these novel QTSs did not exhibit a large effect size and were likely suppressed by some other QTSs associated with the other traits.

### Bioinformatics analysis for candidacy of genes

According to the latest version of rice genome annotation (MSU Rice Genome Annotation Project Release 7, http://rice.plantbiology.msu.edu/index.shtml), of the 39 significantly QTSs detected for yield traits from the unconditional and conditional mapping, 18 QTSs were located within known annotated genes (Table 5). Five of these were identified simply as genes encoding hypothetical proteins or expressed proteins. The remaining genes were found to encode particular enzymes, domains, transcription factors, and transposon or retrotransposon proteins, which might function in important roles in plant development. For instance, the LOC_Os06g43680 gene harboring the QTS rs26302731 associated with grain yield (detected both by conditional and unconditional mapping) putatively encodes the palmitoyltransferase TIP1, which was revealed by prior studies^16, 17^ to be involved in a number of processes including root hair and pollen tube growth in Arabidopsis. Another conditional QTS, rs3063720, is located in the LOC_Os10g06030 gene. This gene encodes a rice wall-associated kinase (OsWAK) that is known to play a critical role in communication between the plant cell wall and the cytoplasm^18^ and has been shown via functional studies in Arabidopsis to be involved in various functions such as cell expansion^19, 20^. The QTS rs28989077 associated with NP is located in the LOC_Os03g50780 gene, which encodes a protein containing a PHD finger domain. PHD finger domains are thought to play an important role in the regulation of chromatin or transcription^21, 22^. SNP rs20829501 is another NP-associated QTS in the LOC_Os07g34770 gene. A previous study^23^ reported that the function of LOC_Os07g34770 may relate to rice seed dormancy, but evidence for its possible association with NP is, to our knowledge, new in the literature. In addition, the rest of the detected QTSs that are not located in known genes but they are near some sensible genes. For instance, rs41315645, found in our study to be related to filled-grain numbers, is located ~12 kb downstream of the LOC_Os01g71310 gene, which exhibits the same gene function as the known cloned gene *Gn1a* (MSU: LOC_Os01g10110, http://www.ricedata.cn/gene/). *Gn1a* encodes a cytokinin oxidase/dehydrogenase that degrades bioactive cytokinins; and reduced expression of *Gn1a* can lead to the accumulation of cytokinins and thus increase the number of grains^24^.

**Table 5:** Detected significant SNPs located within annotated genes.

QTS	Chr.	Allele	Trait	Gene ID	Gene Annotation
rs5137246	6	T/G	GYD	LOC_Os06g10090	hypothetical protein
rs17926420	12	C/A	GYD,GYD|NFGP,GYD|GW	LOC_Os12g29990	O-sialoglycoprotein endopeptidase, putative, expressed
rs26302731	6	G/A	GYD,GYD|NP	LOC_Os06g43680	palmitoyltransferase TIP1, putative, expressed
rs15874222	2	T/A	GYD|GW	LOC_Os02g27000	ATP-binding region, ATPase-like domain containing protein, expressed
rs28567251	3	T/C	GYD|NFGP	LOC_Os03g50090	transposon protein, putative, CACTA, En/Spm subclass, expressed
rs28377293	6	G/C	GYD|NFGP	LOC_Os06g46720	retrotransposon protein, putative, unclassified, expressed
rs28331893	7	A/C	GYD|NFGP	LOC_Os07g47360	CW-type Zinc Finger, putative, expressed
rs11756284	2	T/G	GYD|NP	LOC_Os02g19980	expressed protein
rs5137241	6	T/C	GYD|NP	LOC_Os06g10090	hypothetical protein
rs3063720	10	G/A	GYD|NP	LOC_Os10g06030	OsWAK103 - OsWAK receptor-like protein kinase, expressed
rs18309603	12	A/G	GYD|NP	LOC_Os12g30500	DUF593 domain containing protein, expressed
rs28989077	3	C/T	NP	LOC_Os03g50780	PHD finger domain containing protein, putative, expressed
rs20829501	7	C/T	NP	LOC_Os07g34770	transposon protein, putative, CACTA, En/Spm subclass, expressed
rs9429313	10	A/C	NP	LOC_Os10g18590	retrotransposon protein, putative, unclassified, expressed
rs27878540	3	C/T	NFGP	LOC_Os03g48950	expressed protein
rs29922937	3	A/T	NFGP	LOC_Os03g52110	retrotransposon protein, putative, Ty3-gypsy subclass, expressed
rs12778614	2	A/T	GW	LOC_Os02g21530	expressed protein
rs18572583	3	T/C	GW	LOC_Os03g32480	retrotransposon protein, putative, unclassified, expressed

Note: Gene annotation information comes from the database: MSU Rice Genome Annotation Project Release 7, http://rice.plantbiology.msu.edu/index.shtml.

## Discussion

In the last decade, genome-wide association method have been a primary tool for dissecting complex traits, especially for human diseases. Such methods have also become appealing and affordable in plant research programs owing to dramatically-reduced costs for genomic technology services. Even though genome-wide association studies (GWASs) have led to some promising scientific discoveries, they have encountered the ‘missing heritability’ problem. This refers to the situation where identified genetic variants (mainly SNPs) only explain a small proportion of the expected heritability estimated from classical pedigree analyses. It has been suggested that the failure to evaluate genetic interactions (epistasis, gene-by-environment interactions) is a reasonable explanation for this phenomenon^25, 26^. In this study, a saturated model based on a mixed linear model approach was adopted to identify additive, additive by additive epistasis, and their interactions with environment effects simultaneously. Gene-by-environment interactions were detected for GYD (Table 3), which might partly account for the significant differences for mean of grain yield in the two environments that we observed in our phenotypic analysis (Table 1). There was also one pair of epistasis detected separately for NP and NFGP, which is consistent with the inference from the phenotypic distribution analysis that the non-normal distribution implied the existence of non-additive effects. Even though the genetic interactions did not contribute to a large degree of heritability (~2.0%) in this study, a relatively large total heritability (~45%) was observed for each trait. This large heritability might result from our use of controlled experimental population for association mapping, which compromised the resolution to some extent whereas acquired the advantage of well-controlled population structure and thus increased the explainable heritability. In addition, this study based on the RIL population derived from Xieyou9308 can only reveal part of the genetic basis for its high yield potential because all the RIL lines are theoretically homozygous genotypes and it can hardly address the genetic basis for heterosis which mainly rises from heterozygotes. Thus, for further investigation of the genetic basis for heterosis, the immortalized F_2_ (IF_2_) population that is generated from random mating of recombinant inbred (RI) strains would be ideal, since it contains more heterozygous loci as well as more kinds of combination of genes in different positions on genome which are basic requirements for analyzing dominance, dominance-related epistasis effects.

Conditional analysis is another important tool used to increase the extent of explainable heritability in GWAS by identifying additional secondary association signals conditioned on the primary associated signals^27–29^. Here, we adopted this methodology to analyze the genetic interrelationships between rice yield and its three components, and further detected some additional QTSs for final yield by conditioning on its component traits. Grain yield in rice can be viewed as the integration of some quantitative component traits, which, as proposed by Piepho^30^, can be represented by observed primary characters like number of panicles (NP), number of filled grains (NFGP) and grain weight (GW). This integrated character also complicates the causal gene mapping for final grain yield, especially because of the negative correlations between the components. Thus, it has been thought more effective to dissect its component traits individually, as these probably have simpler genetic control and can exclude the influence of the other components. In this study, we first performed genome-wide association mapping for all four traits separately. There were a total of 24 significant unconditional QTSs detected and the two or three highly significant QTSs for each trait were found to be located within or near annotated genes, most of which are predicted to have functions conceivably associated with target traits (Table 3, Table 5). Although quite high phenotypic correlations were observed between grain yield and some of its components (NP and NFGP), there were no coincident QTSs detected for grain yield and its components. Conditional analysis was conducted to complement our understanding of the relationships between the causal and resultant traits and to reveal some novel candidate loci for yield traits. We found 21 SNPs that significantly affected yield, among which 6 were revealed by the unconditional mapping and 19 by the conditional mapping (Table 4). SNP rs8203251 on chromosome 4 was the only QTS for yield that could be detected without the influence of any individual component. The QTSs rs5137246 and rs12354751 were undetectable in the conditional mapping, which indicates the close correlation between these two loci and yield component traits. 3 QTSs (rs26302731, rs17926420, and rs26662491) could be detected both in conditional and unconditional mapping; the remaining 15 were novel conditional QTSs; 8 of them were located within annotated genes. These results suggested that conditional mapping can help to identify more QTSs for grain yield.

As for practical breeding, we detected some high potential candidates for these yield traits. For grain yield, rs26302731 appears to be a reliable candidate locus due to its high heritability (*h* ^2^ = 9.34%) and corresponding gene functional analysis in Arabidopsis, which revealed its regulation role for plant cell growth^16^. Additionally, the conditional mapping analysis indicated that rs26302731 is supposed to be independent of the panicle number trait. The QTS rs8203251 is another reliable candidate locus, although it was not located in an annotated gene. It is quite special; it is the only QTS found to be independent of all three components and to exhibit a very high heritability (*h* ^2^ = 10.91%). The nearest gene to it was LOC_Os04g14620 (715 bp away) that encodes a retrotransposon protein belonging to the Ty3-gypsy subclass. Retrotransposons seemed ubiquitous in our results, as they were associated with all four yield traits (the corresponding QTSs are rs9429313 associated with NP, rs29922937 associated with NFGP, rs18572583 associated with GW and rs28377293 associated with GYD conditioned NFGP as showed in Table 5). The QTSs rs28989077 and rs20829501 are two reliable candidate loci for panicle number; both have high individual heritability (*h* ^2^ = 18.54% and *h* ^2^ = 11.39%, respectively) and conceivable gene functions as described in candidate gene analysis section. The QTS rs41315645 is highly correlated with filled-grain number (*h* ^2^ = 8.16%) and near a gene (~12 kb away and there is no SNP in this gene) that exhibits the same function as the cloned gene *Gn1a* which has been demonstrated to have a function in influencing grain number^24^; it should thus be considered a quite reliable candidate for grain number in this population. Novel loci from the conditional mapping also have high potential to be reliable candidates for grain yield along with the analysis of corresponding gene function. For instance, rs3063720 is located in the OsWAK gene; studies in Arabidopsis have shown that this gene functions in cell expansion during plant development^19, 20^ and thus it is a high potential candidate for breeding.

## Methods

### Plant Materials and SNP Genotyping

A recombinant inbred line (RIL) mapping population consisting of 138 F_13_ lines was developed from the super hybrid Xieyou9308 via the single seed descent (SSD) method, with XieqingzaoB (XQZB) (female) as the maintainer line and Zhonghui9308 (ZH9308) (male) as the restorer line. The inbred lines were planted in Lingshui, Hainan province and in Hangzhou, Zhejiang province in 2009, respectively. Four rice yield traits, including grain yield per plant (GYD), panicle number per plant (NP), number of filled-grains per panicle (NFGP), and the weight of 1000 grains (GW) were measured. Based on the mixed linear model approach implemented in the software of QGAStation^31^, the total genotypic values were predicted for the calculation of genetic correlation between traits. Calculations of the summary statistics of the phenotypic data, as well as analysis of the data distributions and correlation coefficients were performed using the R (v3.2.2) statistical software^32^.

DNA re-sequencing was conducted at the Beijing Genome Institute (BGI) for the 138 inbred lines with 2X coverage and the parent lines with 10X coverage. The latest version of the Nipponbare sequence was used as the reference genome. Subsequently, sequence alignment was performed between the re-sequencing data and the reference genome using BWA software^33^. SNPs were identified between the individuals and the reference genome by using SAMtools software^34^, with settings as follows: base quality ≥30, mapping quality ≥20, and the maximum sequence depth ≤1000. Finally, a total of 701,867 SNPs were identified for the 138 RILs and used in the subsequent association studies. The filtration of SNPs and LD pattern analysis have been demonstrated in our another study^35^ based on this RIL population. It has shown that the LD decay rate was estimated approximately 1,000 kb on whole genome-wide. It is noteworthy that associations in this population would not be affected by population structure issues, as these progenies are from the same ancestry (the cross of XQZB and ZH9308), and the relatedness among these lines is distributed evenly (r ≈ 0.5 for any two individuals).

### Genetic Models and Statistical Analysis

In this study, we adopted a saturated genetic model with additive (*a*) and additive by additive epistasis (*aa*) as fixed effects, the environment (*e*) which is mostly uncontrollable as random effects, and thus their interactions (*ae*, *aae*) also as random effects. The genetic model for the phenotypic value of the *k*-th genotype in the *h*-th environment (*y*_*hk*_) can be expressed by the following mixed linear model,

1 $${y}_{hk}=\mu +\sum _{i}{a}_{i}{x}_{ik}+\sum _{i < j}a{a}_{ij}{x}_{ik}{x}_{jk}+{e}_{h}+\sum _{i}a{e}_{hi}{u}_{hik}+\sum _{i < j}aa{e}_{hij}{u}_{hijk}+{\varepsilon }_{hk}$$

Where, *μ* is the population mean; *a*_*i*_ is the additive effect of the *i*-th gene (QTS) with coefficient *x*_*ik*_, fixed effect; *aa*_*ij*_ is the additive by additive epistasis effect of the *i*-th QTS and the *j*-th QTS with coefficient *x*_*ik*_ · *x*_*jk*_, fixed effect; *e*_*h*_ is the main effect of the *h*-th environment, random effect; *ae*_*hi*_ is the additive by environment interaction effect of the *i*-th QTS and the *h*-th environment with coefficient *u*_*hik*_(=*x*_*hik*_), random effect; *aae*_*hij*_ is the interaction effect of the *aa*_*ij*_ and the *h*-th environment with coefficient *u*_*hijk*_(=*x*_*hik*_ · *x*_*hjk*_)), random effect; and *ε*_*hk*_ is the random residual effect of the *k*-th line in the *h*-th environment.

Based on the above mixed linear model, both unconditional and conditional genetic mapping were performed for grain yield and its three components. For conditional association mapping, the conditional phenotypic values of grain yield given its components (T1|T2) were first produced by software of QGAStation 2.0^31^ which implemented the mixed model approach for the conditional analysis of quantitative traits as described by Zhu^13^, where the T1|T2 means trait 1 conditioned on trait 2. GMDR-GPU^36^ was then employed for preliminary filtering for both of the association mapping approaches due to the heavy computational burden resulting from the detection of two-dimensional interactions for a very large number of SNPs. During the process, both the single-locus effects and two-loci interaction effects were tested for each trait in each environment using the GMDR-GPU, and the ~400 top candidate SNPs (setting the “-m 400” option in GMDR-GPU) potentially associated with the trait were kept for each chromosome according to their testing accuracy from high to low. Finally, based on the screened SNP subsets, association mappings were conducted for each trait using the mixed linear approach implemented in QTXNetwork^37^ software. In this procedure, first significant testing for each individual SNP and for all possible SNP pairs were performed using *F*-test which is permutation-based to control the experiment-wise type I error rate at 0.05, and then stepwise model selection was conducted to pick out the relatively high explanatory significant candidates for the final full model (1). Finally, all the parameters and corresponding standard errors of model (1) were estimated via Markov chain Monte Carlo (MCMC) with 20,000 Gibbs sampler iterations. Based on the estimated genetic component effects (additive, epistasis, and their interaction effects with environment), the heritability of each QTS in each genetic component was calculated and the summation of all detected QTSs for the trait is regarded as the total heritability.
